# Molecular epidemiology and antimicrobial resistance patterns of carbapenem-resistant *Acinetobacter baumannii* isolates from patients admitted at ICUs of a teaching hospital in Zunyi, China

**DOI:** 10.3389/fcimb.2023.1280372

**Published:** 2023-12-01

**Authors:** Lin Xiong, Chengmin Deng, Guangwu Yang, Meijing Shen, Benhai Chen, Rengui Tian, He Zha, Kaifeng Wu

**Affiliations:** ^1^ Department of Laboratory Medicine, The First People’s Hospital of Zunyi (The Third Affiliated Hospital of Zunyi Medical University), Zunyi, China; ^2^ Scientific Research Center, The First People’s Hospital of Zunyi (The Third Affiliated Hospital of Zunyi Medical University), Zunyi, China

**Keywords:** Carbapenem-resistant Acinetobacter baumannii (CRAB), molecular epidemiology, antibiotic resistance, intensive care units (ICUs), multilocus sequence typing(MLST), multiple-locus variable-number tandem repeat analysis(MLVA)

## Abstract

**Background:**

Carbapenem-resistant *Acinetobacter baumannii* (CRAB) has emerged as a predominant strain of healthcare-associated infections worldwide, particularly in intensive care units (ICUs). Therefore, it is imperative to study the molecular epidemiology of CRAB in the ICUs using multiple molecular typing methods to lay the foundation for the development of infection prevention and control strategies. This study aimed to determine the antimicrobial susceptibility profile, the molecular epidemiology and conduct homology analysis on CRAB strains isolated from ICUs.

**Methods:**

The sensitivity to various antimicrobials was determined using the minimum inhibitory concentration (MIC) method, Kirby-Bauer disk diffusion (KBDD), and E-test assays. Resistance genes were identified by polymerase chain reaction (PCR). Molecular typing was performed using multilocus sequence typing (MLST) and multiple-locus variable-number tandem repeat analysis (MLVA).

**Results:**

Among the 79 isolates collected, they exhibited high resistance to various antimicrobials but showed low resistance to levofloxacin, trimethoprim-sulfamethoxazole, and tetracyclines. Notably, all isolates of *A. baumannii* were identified as multidrug-resistant *A. baumannii* (MDR-AB). The *bla*_OXA-51-like_, *adeJ*, and *adeG* genes were all detected, while the detection rates of *bla*_OXA-23-like_ (97.5%), *adeB* (93.67%), *bla*_ADC_ (93.67%), *qacEΔ1-sul1* (84.81%) were higher; most of the Ambler class A and class B genes were not detected. MLST analysis on the 79 isolates identified five sequence types (STs), which belonged to group 3 clonal complexes 369. ST1145^Ox^ was the most frequently observed ST with a count of 56 out of 79 isolates (70.89%). MLST analysis for non-sensitive tigecycline isolates, which were revealed ST1145^Ox^ and ST1417^Ox^ as well. By using the MLVA assay, the 79 isolates could be grouped into a total of 64 distinct MTs with eleven clusters identified in them. Minimum spanning tree analysis defined seven different MLVA complexes (MCs) labeled MC1 to MC6 along with twenty singletons. The locus MLVA-AB_2396 demonstrated the highest Simpson’s diversity index value at 0.829 among all loci tested in this study while also having one of the highest variety of tandem repeat species.

**Conclusion:**

The molecular diversity and clonal affinities within the genomes of the CRAB strains were clearly evident, with the identification of ST1144^Ox^, ST1658^Ox^, and ST1646^Ox^qaq representing novel findings.

## Introduction

CRAB has emerged as a significant contributor to healthcare-associated infections (HAIs) worldwide, particularly in intensive care units (ICUs; Blanco et al., 2018; Busani et al., 2019; Chen et al., 2019). A study conducted by He et al. (2020) revealed a significantly higher rate of sputum isolation of Carbapenem-Resistant *Acinetobacter baumannii* (CRAB) in ICUs compared to non-ICUs. Moreover, there has been an observable upward trend in the resistance rate of CRAB. Similarly, several reports have indicated that sputum specimens account for approximately 80% of CRAB isolates in ICUs, with CRAB constituting over half of all carbapenem-resistant Gram-negative bacilli. Consequently, effectively managing and treating CRAB infections in ICUs poses a global challenge (Jiang et al., 2022). The colonization, infection, transmission, and drug resistance mechanisms of CRAB in ICUs warrant a more systematic investigation (Tomczyk et al., 2019; Jiang et al., 2022). Therefore, comprehensive studies on the molecular epidemiology and genetic characteristics of CRAB in ICUs will serve as a crucial foundation for implementing preventive and control measures against CRAB infections in these settings.

Many variations in antibiotic resistance genes (ARGs) and CRAB molecular typing have been observed across different countries and regions (Liu and Liu, 2021; Wang et al., 2021). In addition to oxacillinase genes (Ambler class D β-lactamases, e.g., *bla*_OXA-23-like_), CRAB isolated from ICUs has also shown the presence of aminoglycoside-modifying enzyme (AME)-encoding genes and *AdeABC* efflux pump genes (Mavroidi et al., 2017; Chen et al., 2018a; Khalil et al., 2021). An increasing number of studies have focused on drug resistance mechanisms and the homology of CRAB in ICUs, particularly in developing nations (Maamar et al., 2018; Zhang et al., 2021; Odih et al., 2022).

The Multilocus sequence typing (MLST) method remains the conventional and widely employed approach for assessing genetic diversity and investigating epidemiology (Dekker and Frank, 2016; Nutman and Marchaim, 2019). It facilitates the tracking of sequence types (STs) and outbreaks while providing insights into microbial evolution (Hammerum et al., 2015). The corresponding cluster analysis based on STs also offers several advantages, including ease of interpretation and the ability to create hierarchical groupings of isolates. Furthermore, it provides a comprehensive overview of isolate relatedness and interconnectivity within defined clusters (Maiden, 2006).

In alternative typing methods, multiple-locus variable-number tandem repeat analysis (MLVA) offers high resolution, data portability, and intra-laboratory reproducibility (Johnson et al., 2016). Both MLVA and MLST approaches enable the identification of clonal lineages and investigation of genetic diversity among clinical *A. baumannii* clones (Hauck et al., 2012).

The primary objective of the present study was to conduct an in-depth analysis of antimicrobial resistance, molecular epidemiology, and phylogenetic relationships among clinical isolates of CRAB obtained from ICUs. This investigation provides foundation for the assessment and control of HAIs and epidemics.

## Materials and methods

### Study site

The study was conducted at the First People’s Hospital of Zunyi (Third Affiliated Hospital of Zunyi Medical University), a university-affiliated teaching hospital located in Zunyi, Guizhou Province, southwestern China. With a capacity of 2500 beds, this hospital annually provides medical care to over 60,000 inpatients and more than 1 million outpatients.

### Bacterial isolates and identification

From September 2018 to the end of July 2021, a total of 79 non-duplicate CRAB isolates were obtained from hospitalized patients at the First People’s Hospital of Zunyi. These clinical specimens were exclusively collected from the respective intensive care units (ICU).

All nonduplicated *A. baumannii* isolates were confirmed using Matrix-Assisted Laser Desorption/Ionization Time of Flight Mass Spectrometry (MALDI-TOF-MS) technology provided by Bio-Mérieux, France.

### Antimicrobial susceptibility testing

A VITEK®2 compact automatic bacterial detection and analysis system (Bio-Mérieux, France) was employed for drug sensitivity testing. Bacterial susceptibility to extended-spectrum cephalosporins (piperacillin, ceftazidime, and cefepime), enzyme-inhibitor complexes (ampicillin/sulbactam, piperacillin/tazobactam, and cefoperazone/sulbactam), aminoglycosides (gentamicin, tobramycin, amikacin), carbapenems (imipenem and meropenem), folate pathway inhibitors (trimethoprim-sulfamethoxazole), fluoroquinolones (ciprofloxacin and levofloxacin), as well as tetracyclines (doxycycline, minocycline, tigecycline) were analyzed in this study. Confirmation tests were conducted using the E-test assays alongside the Kirby-Bauer method.

The strains of *Escherichia coli* (ATCC® 25922), *Pseudomonas aeruginosa* (ATCC® 27853), *Staphylococcus aureus* (ATCC® 25923), *Enterococcus faecalis* (ATCC® 29212) and *Streptococcus pneumoniae* (ATCC® 49619) were procured from the clinical testing center of the National Health Commission for quality control purposes in the antimicrobial susceptibility test. The interpretation of antimicrobial susceptibility results was conducted according to CLSI M100-S31 guidelines, while the MICs of tigecycline were interpreted based on FDA MIC break-point standards available at http://www.fda.gov.

As previously mentioned (Kumburu et al., 2019), CRAB isolates were classified as multidrug-resistant (MDR: non-susceptibility to more than one agent in three or more classes of antibiotics), extensively drug-resistant (XDR: non-susceptibility to one or more agents in all but one or two classes), or pandrug-resistant (PDR: non-susceptibility to representative agents of all classes).

### DNA preparation

The strains were cultured by streaking onto blood agar culture plates and incubated overnight for 16-18 hours. Subsequently, single colonies were transferred to an EP tube containing 1 mL of TE buffer, vigorously shaken, and thoroughly mixed. The mixture was then subjected to heat treatment at 100°C in a water bath for 30 minutes, followed by cooling at -20°C for another 30 minutes. After complete thawing at room temperature, the sample was centrifuged at 12,000 rpm for 10 minutes. Finally, the supernatant (100 µL) was stored at -20°C for further use (Gomaa et al., 2017).

### Identification of genes associated with antimicrobial resistance

The focus of this study is on the analysis of the carbapenem-resistant genome, encompassing class A (*bla*_TEM_, *bla*_KPC-like_, *bla*_GES_, *bla*_SHV_, and *bla*_CTX-M-9_), class B (*bla*_IMP_, *bla*_VIM_, *bla*_NDM_, and *bla*_SIM_), class C (*bla*_ADC_), and class D (*bla_OXA-23-like_, bla_OXA-51-like_, bla_OXA-24-like_, bla_OXA-58-like_*) (Xu and Xu, 2021; Zhu et al., 2022). Various types of resistance genes were selected for analysis, including aminoglycoside resistance gene (*ant* (*3”*)*-I* and *aac* (*3”*)*-I*), Efflux pump gene (*adeB*, *adeG*, *adeJ)*, Disinfectant gene (*qacEΔ1-sul1*), Integron resistance gene (*Intl1*) (Zhou et al., 2020).

Polymerase chain reaction (PCR) testing was used to detect the presence of target genes in the isolates. The PCR products were subjected to analysis using 2% agarose gel electrophoresis followed by sequencing and comparison with sequences in GenBank. The experimental primer sequences were synthesized with positive products being sequenced by BGI tech solutions (BEIJING LIUHE Co., Ltd).

### Multilocus sequence typing

The MLST genotyping was conducted by sequencing seven gene loci (*gltA, gyrB, gdhB, recA, cpn60, gpi*, and *rpoD*) according to the method described previously (Bartual et al., 2005). The PCR products were sequenced by the BGI tech solutions. DNA sequence variations and STs were analyzed using the MLST database for A. baumannii. The Oxford scheme was employed for MLST analysis as described before (https://pubmlst.org/organisms/acinetobacter-baumannii).

ST^Ox^s were grouped into clonal complexes (CCs) if they shared at least 5 identical alleles (single [SLV] or double locus variants [DLV]) with another member of the group using goeBURST online tool (Francisco et al., 2009).

All ST^Ox^s in ZunYi isolates were compared to all ST^Ox^s from China by the goeBURST online for relationship analysis (http://eburst.mlst.net)(Ribeiro-Goncalves et al., 2016).

### Multiple loci VNTR analysis

Based on the MLVA-8 scheme method, each isolate was identified by determining the number of repeats at each VNTR locus. A total of 8 VNTR loci were carefully selected and evaluated using a validation panel consisting of 79 isolates (Pourcel et al., 2011). Oligonucleotide primers specifically targeting the flanking regions of S-repeat VNTRs (Abaum3468, Abaum0845, Abaum0826, and Abaum2396) as well as L-repeat VNTRs (Abaum3,530, Abaum3002, Abaum2240, and Abaum1988) were employed for amplifying genomic DNA from *A. baumannii*. The size of the amplicon was determined using ImageLab v5.2 image analysis software (Bio-Rad, United States). To estimate the number of repeats in VNTR alleles for each isolate, we subtracted the flanking region size from the amplicon size and then divided it by the repeat unit length (Nhu et al., 2014). The numbers of TRs at each locus were combined together to form a unique combination that defined an original MLVA type (MT).

The MLVA clustering of all isolates was performed using the unweighted pair group method with arithmetic mean (UPGMA) in NTsys V2.10e software. A cutoff value of 90% similarity was applied to define clusters, and the MLVA type (MT) was determined using a 100% similarity cutoff. PCR conditions and characteristics of VNTRs were described previously (Nhu et al., 2014; Maleki et al., 2022). The MTs were analyzed using Phyloviz V2.2 software. The clustering of MLVA profiles was conducted using a categorical coefficient (Francisco et al., 2012; Nascimento et al., 2017; Dahyot et al., 2018).

The discriminatory ability of the two typing methods and their combination were evaluated using Simpson’s diversity index (DI)40 with confidence intervals (CI), which were calculated online at http://www.comparingpartitions.info/?link=Tool (Grundmann et al., 2001; Severiano et al., 2011).

## Results

### Epidemiology of CRAB infection

From September 2018 to July 2021, a total of 79 non-duplicated CRAB isolates were isolated from patients, with 25 isolates (31.6%) obtained from females and 54 isolates (68.4%) obtained from males. The mean age of the patients was 58.9 years (range: 0.1-92). These isolates were collected from various clinical specimens, including sputum specimens (69;87.34%), wound secretions (5;6.33%), vascular catheters (2;2.53%), urine samples (2;2.53%), and blood cultures (1;1.27%). All the isolates were isolated from eight ICUs: branch intensive care unit (Branch ICU) accounted for only two cases (2/79 = 2·5%); cardiopulmonary intensive care unit (CPICU), one case (1/79 = 1.3%); emergency intensive care unit (EICU), nine cases (9/79 = 11.4%); general intensive care unit (ICU), twenty-three cases (23/79 = 29.1%); neurological intensive care unit (NICU), thirty-two cases (32/79 = 40.5%); pediatric intensive care unit (PICU), three cases (3/79 = 3.8%); respiratory intensive care unit (RICU), four cases (4/79 = 5.1%); surgical intensive care units (SICUs) had five cases in total (5/78 = 6.3%).

### *In vitro* antimicrobial susceptibility

The results demonstrated that tigecycline exhibited a sensitivity rate of 93.67% (MICs ranging from 0.25 to 2 μg/ml), while all non-sensitive *A. baumannii* isolates displayed MICs in the range of 4 to 8 μg/ml for tigecycline. Additionally, a resistance rate of 100% was observed for piperacillin, Ampicillin/sulbactam, imipenem, gentamicin, ciprofloxacin, ceftazidime, and piperacillin/tazobactam. Furthermore, the non-sensitivity rates to other antibiotics were ≥ 56.96%, with cefepime (100%), cefoperazone/sulbactam sodium (100%), levofloxacin (100%), doxycycline (97.47%), meropenem (94.94%), amikacin (93.67%), tobramycin (91.14%), trimethoprim-sulfamethoxazole (73.42%) and minocycline(56.96%) being notable examples as shown in **Table 1**. The intermediary and sensitivity rates of tigecycline were found to be 2.53% and 93.67%, respectively, based on antibiotic susceptibility testing results. Out of the 79 CRAB isolates screened, all were identified as MDR.

**Table 1:** The antimicrobial resistance profiles of 79 carbapenem-resistant *Acinetobacter baumannii* (CRAB) isolates.

Antibiotics	Number of detected isolates [n (%)]			Interpretive categories and MIC breakpoints (μg/ml)			MICs range (μg/ml)
	Resistance	Intermediary	Sensitivity	Resistance	Intermediary	Sensitivity	
Penicillin							
Piperacillin	79 (100)	0 (0)	0 (0)	≥128	32-64	≤16	0.5-256
Extended-spectrum cephalosporin							
Ceftazidime	79 (100)	0 (0)	0 (0)	≥14	15-17	≤8	0.12-64
Cefepime	72 (91.14)	7 (8.86)	0 (0)	≥14	15-17	≤8	0.12-64
Enzyme inhibitor complex							
Ampicillin/sulbactam	79 (100)	0 (0)	0 (0)	≥32/16	16/8	≤8/4	8/4-32/16
Piperacillin/tazobactam	79 (100)	0 (0)	0 (0)	≥128/4	32/4-64/4	≤16/4	2/4-128/8
Cefoperazone/sulbactam	60 (75.95)	19 (24.05)	0 (0)	≥64	16-32	≤8	8-64
Aminoglycosides							
Gentamicin	79 (100)	0 (0)	0 (0)	≥16	8	≤4	1-16
Tobramycin	70 (88.61)	2 (2.53)	7 (8.86)	≥16	8	≤4	1-16
Amikacin	73 (92.41)	1 (1.27)	5 (6.33)	≥64	32	≤16	2-64
Carbapenems							
Imipenem	79 (100)	0 (0)	0 (0)	≥8	4	≤2	0.25-16
Meropenem	74 (93.67)	1 (1.27)	4 (5.06)	≥8	4	≤2	0.25-16
Folate pathway inhibitors							
Trimethoprim-sulfamethoxazole	58 (73.42)	0 (0)	21 (26.58)	≥2/38	–	≤4/76	1/19-16/304
Fluoroquinolones							
Ciprofloxacin	79 (100)	0 (0)	0 (0)	≥4	2	≤1	0.25-4
Levofloxacin	31 (39.24)	48 (60.76)	0 (0)	≥8	4	≤2	0.5-16
Tetracyclines							
Doxycycline	77 (97.47)	0 (0)	2 (2.53)	≥16	8	≤4	0.5-16
Minocycline	21 (26.58)	24 (30.38)	34 (43.04)	≥16	8	≤4	1-16
Glycylcyclines							
Tigecycline	3 (3.80)	2 (2.53)	74 (93.67)	≥8	4	≤2	0.5-8

### The prevalence of drug resistance genes

The *bla_OXA-51-like_*, *adeJ*, and *adeG* genes were detected in all 79 CRABs. The detection rates of other drug resistance genes were as follows: *bla_TEM_* (64.56%), *bla_ADC_* (93.67%), *qacEΔ1-sul1* (84.81%), *bla_OXA-23-like_* (97.5%), *intl1* (72.5%), *ant (3”)-I* (62.03%), *aac (3”)-I* (58.23%), *adeB* (93.67%), as presented in **Table 2**. The majority of the genes belonging to class A and class B were not detected in the samples analyzed in this study. Analysis results obtained from GenBank revealed consensus rates ranging from 98% to 100%.

**Table 2:** Detection of main drug resistance genes on 79 isolates of CRAB.

Resistance genes	Number (total79)	Rate (%)
Class A		
*bla*_TEM_	51	64.56%
*bla*_KPC-_ *_like_*	0	0
*bla*_GES_	0	0
*bla*_SHV_	0	0
*bla*_CTX-M-9_	0	0
Class B		
*bla*_IMP_	0	0
*bla*_VIM_	0	0
*bla*_NDM_	0	0
*bla*_SIM_	0	0
Class C		
*bla*_ADC_	74	93.67%
Class D		
*bla*_OXA-23-_ *_like_*	77	97.50%
*bla*_OXA-51-_ *_like_*	79	100.00%
*bla*_OXA-24-_ *_like_*	0	0
*bla*_OXA-58-_ *_like_*	0	0
Disinfectant gene		
*qacEΔ1-sul1*	67	84.81%
Integron resistance gene		
*Intl1*	55	69.62%
Aminoglycoside resistance gene		
*ant (3”)-I*	49	62.03%
*aac (3”)-I*	46	58.23%
Efflux pump gene		
*adeB*	74	93.67%
*adeG*	79	100.00%
*adeJ*	79	100.00%

### MLST genotyping

**Figure 1:**
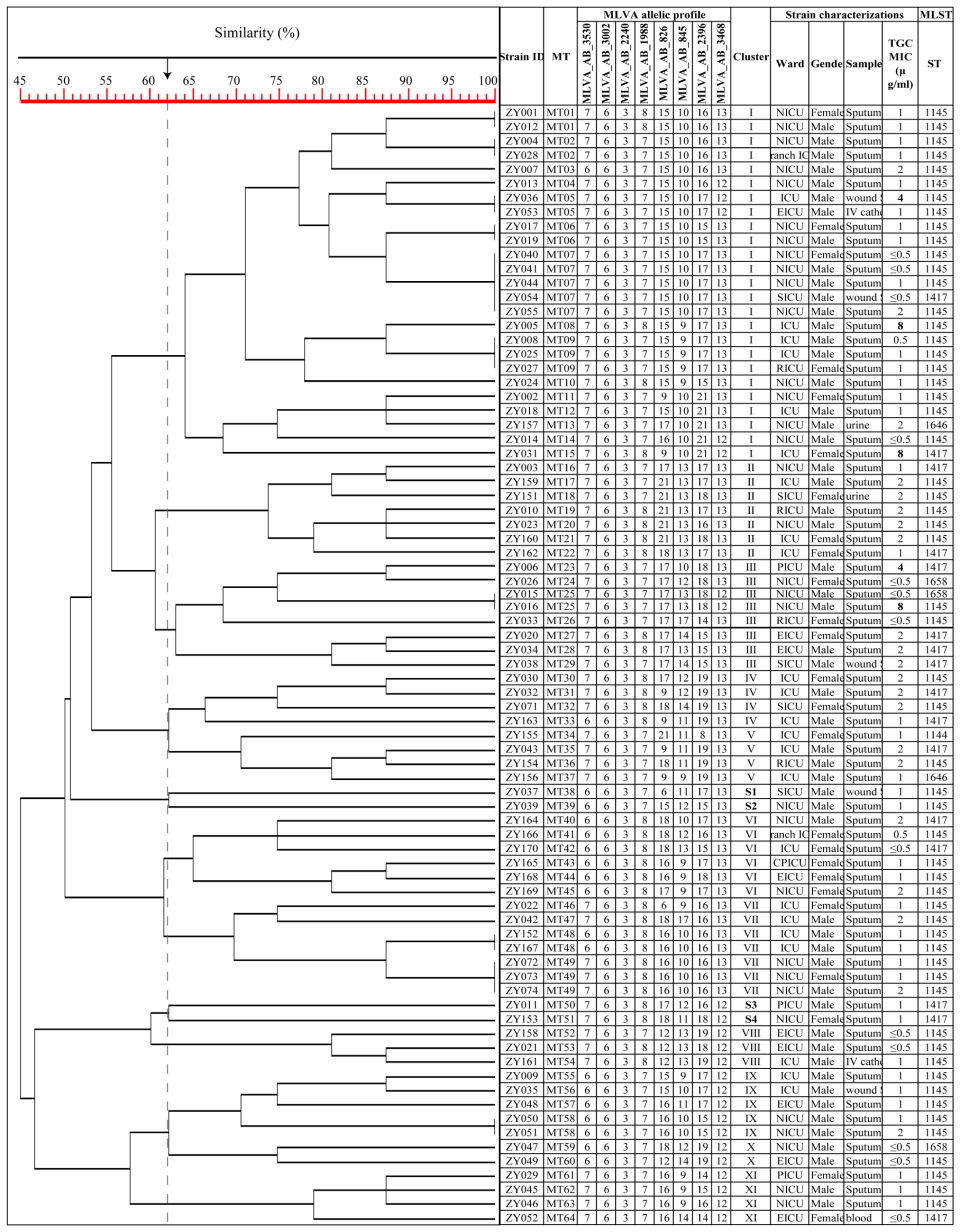
Dendrogram of the genetic diversity among 79 CRAB isolates. ZY 1, isolate number 1; S1, singleton 1; TGC, tigecycline; MIC, minimum inhibitory concentration; The MLVA allelic profile represents each isolate’s number of repeats in the VNTR allele.

**Figure 2:**
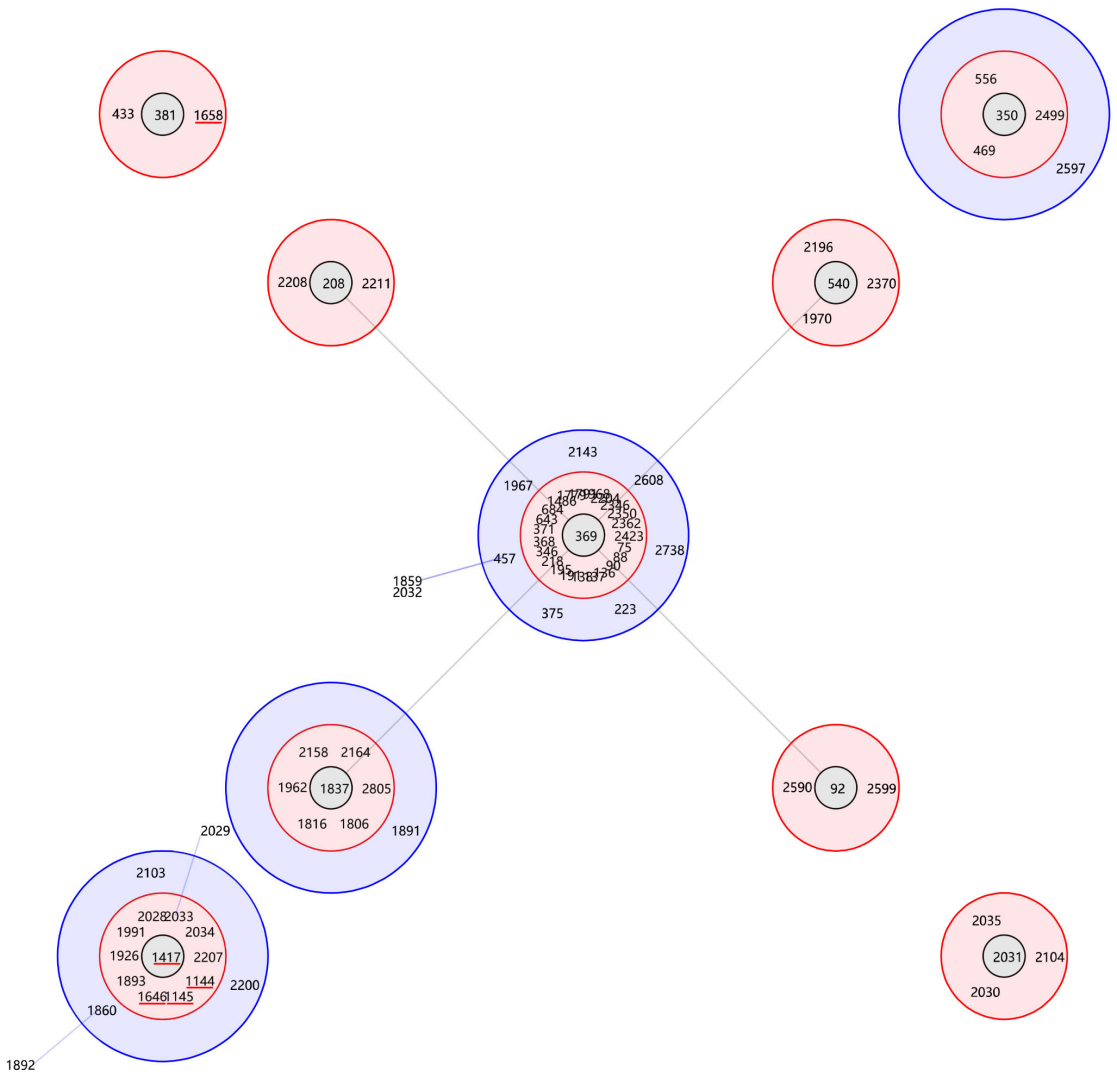
Genetic relationship of the majority *A. baumannii* isolates in China. The genetic relationship of the majority *A. baumannii* isolates in China was analyzed using goeBURST, highlighting 5 specific sequence types (STs) indicated by the red line.

The MLST analysis of the 79 isolates revealed the presence of 5 distinct sequence types (STs). Subsequently, the sequencing results for these 79 isolates were uploaded to the database and assigned accession numbers ranging from 6163 to 6241 (https://pubmlst.org/organisms/acinetobacter-baumannii). ST1145^Ox^ was the most frequently ST (56, 70.89%), followed by ST1417^Ox^ (17, 21.52%), ST1658^Ox^ (3, 3.80%), ST1646^Ox^ (2, 2.53%) and ST1144^Ox^ (1, 1.27%). MLST analysis identified ST1145^Ox^ and ST1417^Ox^ among the isolates with non-sensitive tigecycline, As shown in **Figure 1** (Maleki et al., 2022). A minimal spanning tree, encompassing 55 groups and 129 individual instances, was constructed based on all sequence types (STs) in China. These five STs are classified within group 3, clonal complex 369 (CC369), which represents the largest cluster (**Figure 2**). Specifically, four STs (ST1144^Ox^, ST1145^Ox^, ST1417^Ox^, and ST1646^Ox^) were grouped under branch CC1417, while ST1658 was assigned to branch CC381. The GenBank database information provided is accurate as of July 30th, 2023.

The Simpson’s diversity index (DI) was subsequently computed for each of the 7 loci to assess their discriminatory capacity. Among these, the Oxf_gpi locus exhibited the highest DI value (0.455), indicating a greater variety of alleles present. The details are shown in **Table 3**.

**Table 3:** Genetic Characteristics of the CRAB isolates.

	Locus	Tandem Repetition Type	Number of tandem repeats	Simpson’s ID	CI (95%)
Large (L)-repeat VNTRs (MLVA)	MLVA-AB_3530	2	6, 7	0.370	(0.270-0.470)
	MLVA-AB_3002	1	6	0.000	(0.000-0.000)
	MLVA-AB_2240	1	3	0.000	(0.000-0.000)
	MLVA-AB_1988	2	7, 8	0.488	(0.446-0.530)
Small (S)-repeat VNTRs (MLVA)	MLVA-AB_826	8	6, 9, 12, 15, 16, 17, 18, 21	0.827	(0.786-0.868)
	MLVA-AB_845	7	9, 10, 11, 12, 13, 14, 17	0.783	(0.726-0.839)
	MLVA-AB_2396	8	8, 14, 15, 16, 17, 18, 19, 21	0.829	(0.795-0.864)
	MLVA-AB_3468	2	12, 13	0.418	(0.333-0.503)
MLST	Oxf_gltA	1	1	0.000	(0.000-0.000)
	Oxf_gyrB	2	3,81	0.074	(0.153-1.000)
	Oxf_gdhB	1	3	0.000	(0.000-0.000)
	Oxf_recA	1	102	0.000	(0.000-0.000)
	Oxf_cpn60	1	2	0.000	(0.000-0.000)
	Oxf_gpi	5	94, 97, 96, 140, 16	0.455	(0.342-0.567)
	Oxf_rpoD	1	3	0.000	(0.000-0.000)
MLVA		64	MT1-MT64	0.993	(0.986-0.999)
MLST		5	1144,1145,1417,1646,1658	0.455	(0.342-0.567)

DI, Simpson’s diversity index; CI, confidence interval.

### MLVA genotyping and clustering

By employing the MLVA assay, a total of 79 isolates were classified into 64 distinct MTs. Among these, 39 MTs consisted of only one isolate each, while the three predominant MTs were identified as MT7 (n=5), MT9 (n=3), and MT49 (n=3). The level of diversity in the number of VNTRs varied across different VNTR loci. Notably, MLVA-AB_826 exhibited the most significant observed variation in tandem repeat (TR) size, ranging from 6 to 21 repeats. Similarly, the TR size for MLVA-AB_2396 ranged from 8 to 21 repeats, whereas for MLVA-AB_0845 it spanned from 9 to 17 repeats (**Table 3**).

**Figure 3:**
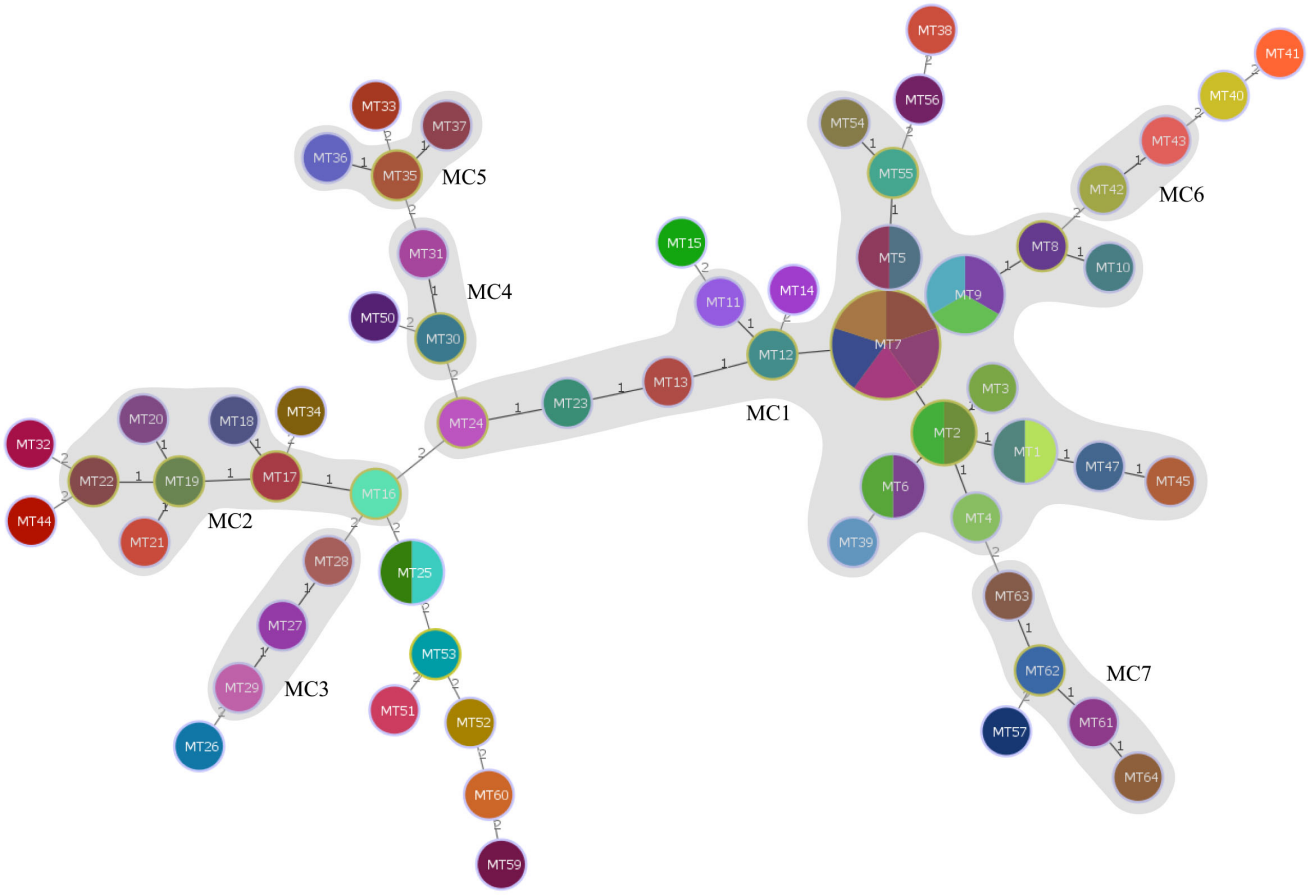
Minimum spanning tree of the 79 CRAB isolates typed by MLVA. The minimum spanning tree was constructed based on the multiple-locus VNTR analysis (MLVA) results of the 79 CRAB isolates using a categorical coefficient. Each circle represents an MLVA type. Gray zones around circles delineate MLVA complexes (MCs). MCs are also indicated in characters; e.g., MC1 denotes MLVA complex 1.

Using the UPGMA algorithm, a total of 11 MLVA clusters (cluster I to XI) and 4 singleton genotypes (S1 to S4) were identified with a cutoff value of 62% similarity. The majority of isolates belonged to cluster I (n=25) and cluster III (n=8), respectively. Additionally, most MTs were classified into cluster I (n=15 MTs; **Figure 1**). For the minimum spanning tree analysis, this study identified a total of 7 distinct MLVA complexes (MCs, MC1 to 7) and 20 singletons (**Figure 3**). Notably, two of these complexes, namely MC1 (comprising 19 MTs and representing 29 isolates) and MC2 (consisting of 7 MTs with 7 corresponding isolates), exhibited significant predominance. Finally, Simpson’s diversity index (DI) was calculated for each of the 8 loci to assess their discriminatory power. Regarding the DI value, MLVA combined 5 loci (MLVA-AB_3530/3002/2240/1988/3468) with low discriminatory power (DI < 0.5), along with 3 highly discriminant locus (MLVA-AB_826/845/2396), in this study. Notably, the MLVA-AB_2396 locus exhibited the highest DI value (0.829), accompanied by one of the greatest numbers of tandem repeats.

## Discussion

CRAB is increasingly emerging as a prominent etiological factor of HAIs among diverse bacteria, particularly in intensive care units (ICUs) (Blanco et al., 2018; Tomczyk et al., 2019; Jiang et al., 2022). Retrospective studies have demonstrated significantly higher rates of CRAB isolation from sputum samples in ICUs compared to non-ICUs, with CRAB accounting for over 50% of carbapenem-resistant Gram-negative bacteria (Lazureanu et al., 2016; He et al., 2020). Over the past two decades, there has been an upward trend in the prevalence of clinical isolates of *A. baumannii* exhibiting CRAB strain characteristics (https://www.chinets.com/Data/GermYear). The present study delineated the resistance spectrum and molecular phenotypes of CRAB in this region exhibited limited variation.

All 79 CRAB isolates showed different resistance patterns to various antimicrobial agents, exhibiting resistance phenotypes to multiple antimicrobial drugs and low resistance rates to tigecycline, with intermediate and susceptibility rates of 2.53% and 93.67%, respectively. These findings are consistent with previous reports from other parts of China (Xu and Xu, 2021; Zhu et al., 2022). However, the drug non-resistance rates of levofloxacin and minocycline were high in this region, 60.76% and 73.42%, respectively. Together with previous reports (Poulikakos et al., 2014; AI et al., 2018), suggesting the possibility of combining carbapenems or other antibiotics with levofloxacin or minocycline may provide a promising treatment option for patients with multidrug-resistant infections.

Numerous mechanisms and genetic determinants contribute to the extensive resistance of CRAB against multiple antibiotics. The predominant mechanisms of drug resistance in CRAB involve the production of Ambler Class C ampC enzyme and Ambler class D oxacillinase enzymes, with *bla*_OXA-23-like_ and *bla*_OXA-51-like_ being the most prevalent genes responsible for class D-mediated CRAB resistance (Qian et al., 2015; Ibrahim, 2019; Say Coskun et al., 2019; Wang et al., 2021). However, no ARGs related to *bla*_OXA-24-like_ or *bla*_OXA-58-like_ were identified, contrasting with several previous reports (Chen et al., 2018b; Kiddee et al., 2018; Ejaz et al., 2021). Despite regional variations in ARG profiles among CRAB strains, both *bla*_OXA-23-like_ and *bla*_OXA-51-like_ hold significant positions as key components.

Meanwhile, no ARGs of ambler class A β-lactamases (*bla_KPC-like_*, *bla_GES_*, *bla_SHV_*, *bla_CTX-M-9_*) and Ambler class B metallo-ß-lactamases (*bla_IMP_*, *bla_VIM_*, *bla_NDM_*, *bla_SIM_*) were detected except for the presence of *bla_TEM_* (64.56%). TEM-type β-lactamases belong to broad-spectrum enzymes that primarily hydrolyze third-generation cephalosporins and their encoding genes are commonly located on the TNL sequence of plasmid transposons which exhibit frequent horizontal transfer events. Moreover, international reports indicate carrier rates around 30% (Maamar et al., 2018; Odih et al., 2022).

Currently, qacEΔ1-sull is one of the most prevalent genes in the qac gene family of disinfectant resistance genes (Hamidian and Nigro, 2019), and the detection rate of qacEΔ1-sull in this study was 84.81%. It is through the action of integrons (e.g., intl1) that the qacEΔ1-sull gene can be transmitted between AB strains, but the detection rate of the qacEΔ1-sull gene is lower than that of the integron resistance gene, also reflecting that this is not the only pathway. Also, the qacEΔ1-sull gene can exhibit resistance by associating the expression of multiple efflux pump genes within the pathogenic bacteria. The mechanism of CRAB resistance is very sophisticated. In addition to the production of β-lactamases, there are mechanisms such as reduction of outer membrane permeability, alteration of drug targets, overexpression of efflux pumps, and biofilm production. As (Mavroidi et al., 2017; Chen et al., 2018a; Khalil et al., 2021), research on biofilm and efflux pump genes of CRAB strains isolated from ICUs has been intensified, and related studies have gradually gained importance; reports also indicate that high level of expression of efflux pump genes has a significant impact on the expression of drug-resistant phenotypes of A. baumannii.

Tigecycline has emerged as one of the last resort antibiotics for treating CRAB infections in ICUs due to the increasingly limited options available (Lin et al., 2020). A notable phenomenon is the emergence of CRAB strains with a G variant mutation that confers resistance to tigecycline (Maleki et al., 2022), indicating potential changes in the population structure of *Acinetobacter baumanni*i. An increasing number of researchers are integrating CRAB’s resistance mechanisms and homology into their investigations (Jiang et al., 2022). Literature details reveal that most studies on the molecular characterization of CRAB in intensive care units (ICUs) have been conducted in developing countries. Variations in antimicrobial resistance genes (ARGs) and molecular typing of CRAB exist across different countries, regions, general wards, and ICUs.

In this current study, all tigecycline non-susceptible strains were exclusively classified under the predominant ST1145^Ox^ and ST1417^Ox^. The presence of *A. baumannii* ST1145^Ox^ has been documented in Qingyuan, Guangdong, China (Xu et al., 2020), but all isolates belong to non-CRAB strains and tigecycline susceptible strains. The ST1145^Ox^ CRAB strains, in contrast, was exclusively identified during a CRAB outbreak within the acute care area of a general hospital in Hong Kong, China (Lee et al., 2022). The occurrence of these two reported cases in such close proximity is remarkably significant. This phenomenon is perhaps characterized by a particular geographical feature precisely because of the proximity of this territory to the two places mentioned above as well. The only documented case of ST1417^Ox^ CRAB originating from humans was identified in Thailand, with the isolation of just two strains (Singkham-In and Chatsuwan, 2018).

More to the point, the present study represents the first report of ST1144^Ox^, ST1658^Ox^, and ST1646^Ox^ isolates of *A. baumannii*, as no previous records were found in the PubMLST database for these types of CRAB.

Clonal relation analysis using eBURST revealed that these five STs were grouped in clonal complex 369 (CC369), with four of them (ST1144^Ox^, ST1145^Ox^, ST1417^Ox^, and ST1646^Ox^) clustered in clade CC1417 while ST1658^Ox^ belonged to clade CC381. Notably, Clades CC1417 and CC381 significantly differed from the predominant clades CC92 and CC208 in China, there were significant genetic distances between the two clades and the dominant prevalent clades CC92, CC195, CC208, and CC95 in China (Qian et al., 2015; Zhou et al., 2015; Guo and Li, 2019; Liu and Liu, 2021; Wang et al., 2021). The previous three were all part of group 3 while CC95 was a separate singleton. Additionally, it is worth noting that CC92 was also predominant in Asia (Kim et al., 2013).

The typing ability, reproducibility, and epidemiological concordance of both MLVA and the widely used MLST were excellent for comparison. However, despite measuring different evolutionary mechanisms at multiple genomic loci, similar clustering was obtained using both methods. Therefore, our results suggest that MLVA-targeted loci provide a reliable measure of genetic relationships. Importantly, MLVA exhibits more differential features in terms of the number of profiles and discriminant index compared to MLST. This is likely due to the faster evolution of VNTRs compared to housekeeping genes, allowing for more sensitive expression of strain evolutionary differences. The higher discriminant index of MLVA enables further differentiation within MLST-defined clonal complexes (CCs) and among clonal lines with significant genotypic diversity. Previous studies have described this phenomenon in *Staphylococcus lugdunensis* (Dahyot et al., 2018), *Listeria monocytogenes* (Chenal-Francisque et al., 2013), and *Streptococcus agalactiae* (Haguenoer et al., 2011). These findings suggest that MLVA may be useful in identifying hospital outbreaks.

Looking at the details of the typing results, we found that the differences in each of the two typing results were concentrated in a few individual loci, such as MLST-AB_gpi and MLVA-AB_826/845/2396. This characteristic has also been demonstrated in our other study on carbapenem-resistant *Klebsiella pneumoniae* (Shen et al., 2023). The above phenomena suggested that the local carbapenem-resistant bacteria have a relatively unique and concentrated molecular genetic profile. MLST and MLVA techniques were employed for molecular typing and evolutionary analysis, providing a comprehensive assessment of the relatedness among the isolated and interconnected clusters. Monitoring bacterial resistance phenotypes combined with bacterial homology analysis is crucial in guiding antibiotic usage for treating *A. baumannii* infections and controlling drug resistance prevalence.

## Conclusion

The resistance profiles and molecular phenotypes of CRAB strains in this region are relatively concentrated. STs were distinctly geographically characterized, and ST1144^Ox^, ST1658^Ox^, and ST1646^Ox^ sequence types for *Acinetobacter baumannii* are reported for the first time, all belonging to CC365. The typing difference loci were all clustered at individual loci, such as MLST_gpi and MLVA-AB_826/845/2396.

Even more importantly, MLST typing provides an excellent means to analyze strain homology across wider geographical areas while introducing MLVA typing allows for sharper distinction between local isolate homologies essential for identifying hospital outbreaks. Combining the two methods demonstrated higher accuracy, reproducibility, timeliness, and cost-effectiveness.

## Data availability statement

The datasets presented in this study can be found in online repositories. The names of the repository/repositories and accession number(s) can be found below: https://pubmlst.org/bigsdb?db=pubmlst_abaumannii_isolates, 6163 to 6241.

## Ethics statement

The studies involving humans were approved by The Ethics Committee of the First People’s Hospital of Zunyi. The studies were conducted in accordance with the local legislation and institutional requirements. Written informed consent for participation was not required from the participants or the participants’ legal guardians/next of kin in accordance with the national legislation and institutional requirements.

## Author contributions

LX: Conceptualization, Data curation, Writing – original draft, Project administration. CD: Writing – original draft, Formal Analysis, Methodology. GY: Methodology, Writing – review & editing. MS: Data curation, Methodology, Writing – review & editing. BC: Data curation, Methodology, Writing – review & editing. RT: Data curation, Methodology, Writing – review & editing. HZ: Funding acquisition, Writing – review & editing. KW: Conceptualization, Funding acquisition, Writing – review & editing.
